# Links between High-Sensitivity C-Reactive Protein and Pulse Wave Analysis in Middle-Aged Patients with Hypertension and High Normal Blood Pressure

**DOI:** 10.1155/2019/2568069

**Published:** 2019-07-17

**Authors:** Ioana Mozos, Daniela Jianu, Cristina Gug, Dana Stoian

**Affiliations:** ^1^Department of Functional Sciences, “Victor Babes” University of Medicine and Pharmacy, 300173 Timisoara, Romania; ^2^Center for Translational Research and Systems Medicine, “Victor Babes” University of Medicine and Pharmacy, 300173 Timisoara, Romania; ^3^1st Department of Internal Medicine, “Victor Babes” University of Medicine and Pharmacy, 300041 Timisoara, Romania; ^4^Military Hospital, 300041 Timisoara, Romania; ^5^Department of Microscopic Morphology, “Victor Babes” University of Medicine and Pharmacy, 300041 Timisoara, Romania; ^6^2nd Department of Internal Medicine, “Victor Babes” University of Medicine and Pharmacy, 300723 Timisoara, Romania

## Abstract

Arterial stiffness and arterial age provide valuable prognostic cardiovascular information. The present study aimed at assessing the levels of vitamin D, high-sensitivity C-reactive protein (hsCRP), low-density lipoprotein cholesterol (LDL), and oxidized LDL (oxLDL) in a group of middle-aged hypertensive patients and their relationship with pulse wave velocity (PWV), central blood pressure, and early arterial aging (EAA), respectively. A total of 56 patients, aged 48 ± 6 years, 57% males, with hypertension and high normal blood pressure (HNBP), were investigated using a Mobile-O-Graph, to assess central and peripheral blood pressure, PWV, and arterial age. Additionally, hsCRP, LDL, oxLDL, and 25-hydroxy vitamin D3 were assessed. PWV, 25-hydroxy vitamin D3, hsCRP, oxLDL, and LDL levels were 7.26 ± 0.69 m/s, 25.99 ± 11.17 microg/l, 0.48 ± 0.44 mg/dl, 261.37 ± 421 ng/ml, and 145.73 ± 39.53 mg/dl, respectively. Significant correlations were obtained between oxLDL and pulse pressure amplification (rS = −0.347, *p* = 0.028) and between hsCRP and LDL levels with PWV and EAA, respectively. ROC curve analysis revealed that hsCRP is a sensitive and specific predictor of EAA and increased PWV values. Concluding, vitamin D deficiency and increased hsCRP and LDL values are very common, and high oxidized LDL is related to pulse pressure amplification in patients with elevated blood pressure. Vitamin D level and high-sensitivity C-reactive protein and LDL provide valuable information in middle-aged hypertensive and HNBP patients related to arterial stiffness and early arterial aging, but only hsCRP is a sensitive predictor of EAA and PWV.

## 1. Introduction

Cardiovascular disorders are leading causes of death worldwide, and Romania was considered a high-cardiovascular-risk country according to the guidelines of the European Society of Cardiology [1]. Early detection of cardiovascular risk and subclinical atherosclerosis should represent a priority, along with the detection of new cardiovascular active substances. Arterial stiffness and arterial age provide valuable prognostic cardiovascular information [2]. Arterial stiffness, the expression of impaired arterial elasticity, may be assessed using pulse wave velocity (PWV) or the augmentation index (AI). AI is a wave reflection parameter and an indirect biomarker of arterial stiffening [3, 4], while arterial age represents the chronological age of a person with all risk factors at normal levels and the same 10-year predicted risk [5].

Vitamin D is essential not just for musculoskeletal health, considering that low 25-hydroxy vitamin D levels were frequently associated with an increased risk of cardiovascular events, including stroke and heart failure [6–9]. C-reactive protein, an acute phase reactant and a marker of chronic low-grade inflammation, can predict cardiovascular events and was mentioned as the only circulating biomarker related to vascular wall biology [3, 10, 11]. High-sensitivity CRP (hsCRP) is a better predictor of vascular disorders than CRP. LDL cholesterol and oxidized LDL favor the formation of the foam cells and the development of the atherosclerotic plaque. There is a continuous relationship between blood pressure and cardiovascular events, and diagnosis of hypertension also requires evaluation of cardiovascular risk factors.

Considering the importance of prophylactic measures in hypertension, the present study aimed at assessing the levels of several laboratory biomarkers such as vitamin D, high-sensitivity C-reactive protein, LDL, and oxidized LDL (oxLDL) and their impact on pulse wave velocity, central blood pressure, and early arterial aging (EAA), respectively, in a group of middle-aged patients with hypertension and high normal blood pressure.

## 2. Materials and Methods

### 2.1. Study Population

A total of 56 consecutive hypertensive and HNBP patients, recruited from the Military Hospital Timisoara, were investigated during the period of July 2016–March 2017. All patients underwent investigation using a Mobil-O-Graph, and biochemical measurements were performed on the same day. Data about cardiovascular risk factors, diagnosis, and therapy were collected from medical records.

Patients aged between 18 and 55 years, with essential hypertension, both treated as well as uncontrolled patients, and high normal blood pressure, were included. Essential hypertension and high normal blood pressure were diagnosed according to the European criteria [12]. Cardiovascular risk in hypertensive patients was evaluated according to the criteria of the European Society of Cardiology, considering the number of cardiovascular risk factors, hypertension-mediated organ damage, and chronic kidney disease [12]. Patients with secondary hypertension, atrial fibrillation, diabetes mellitus, history of coronary heart disease, myocardial infarction, stroke, transient ischemic attack or peripheral arterial disease, systemic inflammatory processes, active infections, trauma, and therapy with statins were excluded.

The investigations conformed to the principles outlined in the Declaration of Helsinki and were approved by the “Victor Babes” University. Written informed consent was obtained from each study participant, and the objectives and procedures of the study were explained to each patient included in the study.

### 2.2. Biochemical Measurements

Blood was drawn after an overnight fast. Serum levels of 25-hydroxy-cholecalciferol, high-sensitivity C-reactive protein, LDL, and oxidized LDL were measured. LDL was assessed using a photometric method, a Siemens Advia 1800 analyzer, and Siemens LDL cholesterol reagents. An ELISA method and Immundiagnostik reagents were used for oxidized LDL. 25-Hydroxy-cholecalciferol and high-sensitivity C-reactive protein were assessed using liquid chromatography and immunophelometry, respectively. The thresholds used for defining normal/abnormal values for the various laboratory variables were provided by the laboratory.

### 2.3. Mobil-O-Graph

Pulse wave velocity (PWV), augmentation index and pressure (AI, AP), arterial (vascular) age, and central and peripheral blood pressure were assessed using a Mobil-O-Graph (IEM GmbH, Stolberg, Germany), a noninvasive, validated device.

The measurements were made after 10 minutes of rest, in supine position, using different cuff sizes, and considering the arm circumference. The participants were not allowed to smoke, eat, or drink coffee or alcohol 4 hours before the recording and speak or move during the measurements.

Mobil-O-Graph enables brachial blood pressure measurement, followed by the reinflation of the cuff and recording of pulse waves, with a high-fidelity pressure sensor [13]. After measuring systolic, diastolic, and mean arterial pressure, the software enabled the reconstruction of the aortic pulse wave using a generalized transfer function [13]. Wave separation analysis enabled decomposing the aortic pulse wave into forward-traveling (incident) and backward-traveling (reflected) pulse waves and allowed calculating the augmentation pressure (AP), augmentation index (AI), and PWV. AP was assessed as the difference in pressure of the reflected wave minus pressure of the forward-traveling wave of the systolic phase of the pulse wave [13]. Central systolic blood pressure (cSYS), central diastolic blood pressure (cDIA), and central pulse pressure (cPP) were also calculated by the device.

AI was defined as the ratio of AP to aortic pulse pressure, indicating the augmentation component of aortic SBP due to the premature arrival of the reflected wave [13]. PWV was estimated from the reconstructed aortic pulse waveform via mathematical models, considering impedance and age [13].

**Figure 1:**
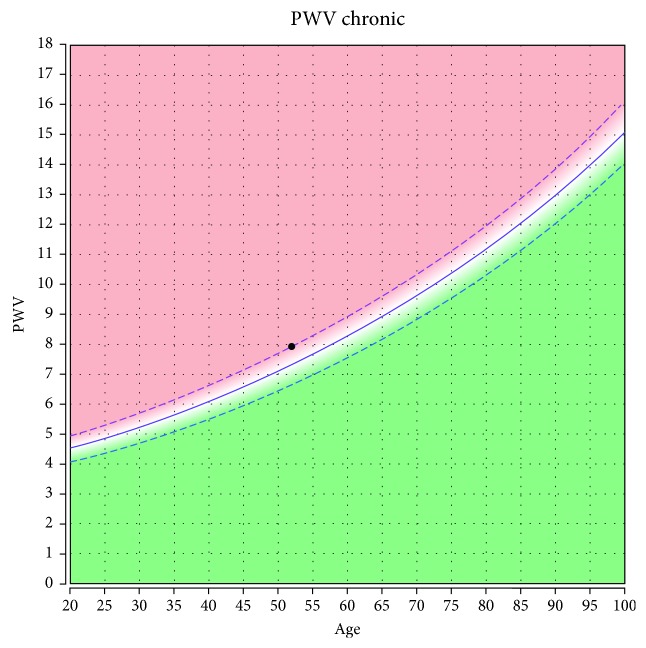
Pulse wave velocity (8 m/s) in a 52-year-old hypertensive patient. Within normal values considering age.

The significance of the variables was also described in previous studies [14]. PWV and AI were considered increased if they exceeded the normal values for the patients' ages according to the device manufacturer, and pathological values were shown by the device (Figure 1). Pulse pressure amplification, defined as cPP/PP [15], was calculated separately. Early arterial aging was considered when arterial age was higher than the biological age.

### 2.4. Statistical Analysis

Categorical data are given as numbers and percentages; continuous data are given as means ± standard deviation. Bravais-Pearson's, Kendall's, and Spearman's correlations and ROC curve analysis were used as statistical methods. Nonparametric correlations were used especially for assessing the correlation between a dichotomous variable and a continuous variable. Analyses were performed using IBM SPSS Base Edition.

## 3. Results

### 3.1. Characteristics of the Study Population

The subjects included in the study were middle-aged (48 ± 6 years), and most of them were male (57%). The baseline characteristics of the study population are included in Table 1.

Vitamin D deficiency and insufficiency, defined as serum levels < 20 microg/l and <30 microg/l, respectively, were very common in the study population, as well as increased hsCRP and LDL values (Table 2). Vitamin D deficiency and insufficiency were defined according to the reference values provided by the laboratory.

### 3.2. Correlations

Correlations were calculated for all variables mentioned in the study objectives, both in a continuous fashion and after categorization. However, no significant correlations were obtained between vitamin D level and pulse wave and blood pressure variables. High-sensitivity C-reactive protein was significantly correlated with early arterial aging, PWV, and pulse pressure amplification (Table 3). LDL cholesterol levels correlated with central systolic and diastolic blood pressure, PWV values, and pulse pressure amplification (Table 3). No significant correlations were obtained between cardiovascular risk (low to very high) assessed according to the criteria of the European Society of Cardiology [12] and vitamin D level, high-sensitivity C-reactive protein, LDL cholesterol levels, and oxidized LDL.

### 3.3. Receiver-Operating Characteristic (ROC) Curve Analysis, Sensitivity, Specificity, and Positive and Negative Predictive Values

**Figure 2:**
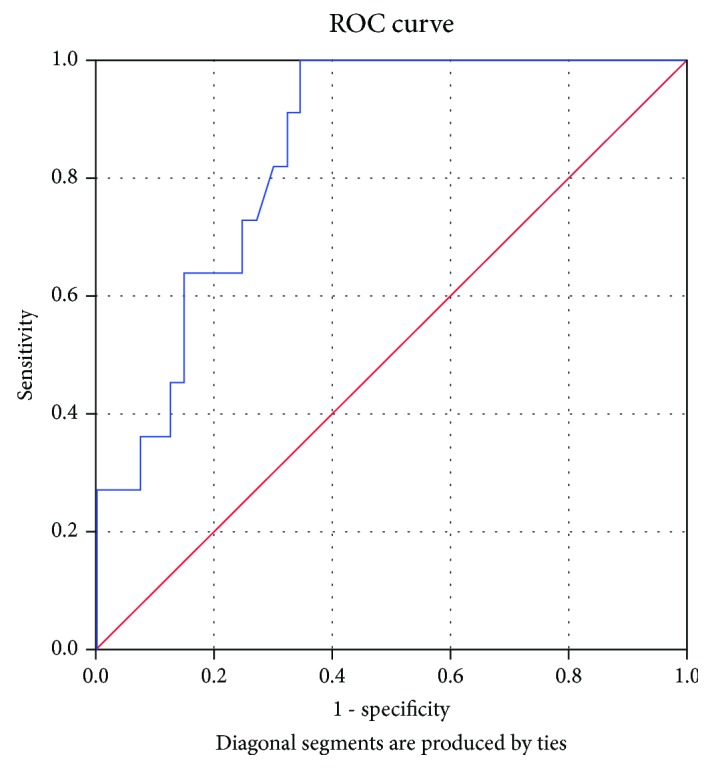
High-sensitivity C-reactive protein as a sensitive and specific predictor of increased pulse wave velocity. AUC = 0.766 (95% CI: 0.603-0.929), *p* = 0.005.

**Figure 3:**
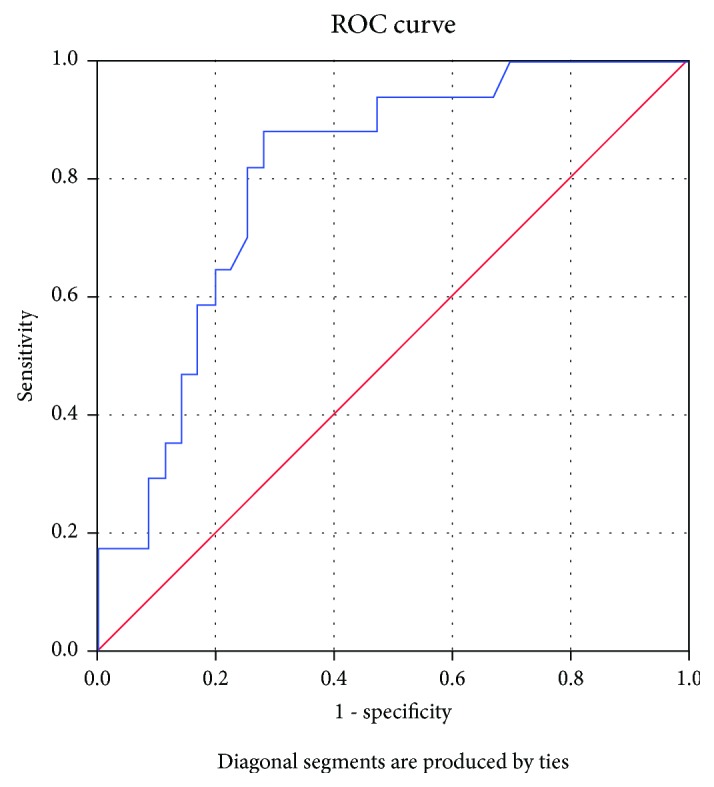
High-sensitivity C-reactive protein as a sensitive and specific predictor of early arterial aging. AUC = 0.749 (95% CI: 0.607-0.890), *p* = 0.003.

ROC curve analysis revealed hsCRP as a sensitive and specific test for EAA and pPWV (Figures 2 and 3). The cut-off value for hsCRP was higher for predicting an increased pulse wave velocity than early arterial aging (Table 4).

Vitamin D, LDL, and oxidized LDL were not revealed as sensitive markers for EAA or PWV according to ROC curve analysis (Table 5).

Further testing revealed important negative predictive values for low vitamin D, oxidized LDL, and hsCRP levels in predicting pathological PWV values and for hsCRP in predicting EAA (Table 6). Oxidized LDL showed good specificity in predicting PWV and EAA, and high-sensitivity C-reactive protein exceeding 0.100 mg/dl, also a good specificity (Table 6).

## 4. Discussion

The present study reports that low vitamin D levels and increased hsCRP and LDL values are very common in middle-aged hypertensive and HNBP patients; correlations were also found between pulse wave velocity and early arterial aging with high-sensitivity C-reactive protein and LDL cholesterol levels, respectively. However, only hsCRP was found as a sensitive predictor of early arterial aging.

Conflicting results have been previously published regarding the relationship between vitamin D level, vitamin D supplementation, and arterial stiffness [16, 17]. Several studies revealed associations between vitamin D level and PWV [18–21], but other authors found no significant association between vitamin D concentrations and markers of subclinical atherosclerosis [22, 23]. The present study found no significant correlation between vitamin D level and PWV but revealed a good negative predictive value of vitamin D level for pathological PWV values. The link between vitamin D deficiency and hypertension can be explained by the activation of the renin-angiotensin-aldosterone system, increasing the vascular tone due to the release of angiotensin II [24]. Vitamin D is also involved in the downregulation of the renin gene expression [25]. Vitamin D has also pleiotropic effects on the immune system and suppresses the low-grade inflammation in the cardiovascular system [17, 26]. Other important pathophysiological vasculoprotective mechanisms by which vitamin D supplementation reduces arterial stiffness include improvement of the endothelial function, suppression of endothelin-induced vascular smooth muscle cell proliferation, effects on calcium metabolism and PTH level, counterbalancing of oxidative stress, and improvement of carbohydrate metabolism and insulin sensitivity [27–33]. Related to oxidative stress, vitamin D activates several genes encoding for antioxidant and detoxifying enzymes [25]. The accumulation of calcium in the vessel walls increases arterial tone and arterial stiffness in hypertension [34]. However, no significant correlations were obtained in our study between vitamin D level and hsCRP or oxidized LDL, respectively.

25-Hydroxy vitamin D and not 1,25-dihydroxy vitamin D was assessed in the present study because 25-hydroxy vitamin D is a nutritional parameter of vitamin D status, the primary circulating and storage form of vitamin D, and a reliable, available marker of low vitamin D levels able to bind to vitamin D receptors [18, 35].

Several mechanisms link inflammation and arterial stiffness, considering the effect of inflammation on the arterial endothelium, nitric oxide (NO), and smooth muscle cells and considering its ability to change the composition of the extracellular matrix, to breakdown elastin, and to enable the calcification of the vessel wall [36]. hsCRP was associated with arterial stiffness in some studies [3, 37–40], but other studies did not find any association [41]. Kim et al. reported significant associations between hsCRP and arterial stiffness independent of age, systolic blood pressure, gender, heart rate, glucose, lipid profiles, and therapy in treated hypertension [38]. The present study found significant correlations and associations between hsCRP and PWV, EAA and pulse pressure amplification, respectively. High-sensitivity C-reactive protein was the only sensitive predictor of early arterial aging and elevated PWV, with a higher cut-off value for predicting increased pulse wave velocity than early arterial aging (0.446 vs. 0.388). Significant correlations were obtained in the present study between hsCRP and LDL130 (exceeding 130 mg/dl) (rK = 0.219, *p* = 0.049; rS = 0.266, *p* = 0.048). High-sensitivity C-reactive protein, a marker of low-grade inflammation, was also associated with deep white matter lesions, ischemic stroke, and heart failure [42, 43]. On the other hand, arterial stiffness was significantly associated with ischemic stroke after adjusting for cardiovascular risk factors [44].

Non-HDL cholesterol was reported as a good predictor of the risk of increased arterial stiffness in postmenopausal women in an urban Brazilian population [45]. Kim et al. reported a modest increase in arterial stiffness due to dyslipidemia only in women [46]. Increased LDL levels correlated with central systolic and diastolic blood pressure, PWV, and pulse pressure amplification in the present study, regardless of gender.

Oxidized LDL was associated with pulse wave velocity in patients with renal failure [47] and in healthy persons between 45 and 69 years old [48]; it was also associated with the augmentation index in obese children and adolescents [49]. In the present study, oxLDL correlated with pulse pressure amplification (rS = −0.347, *p* = 0.028). A high specificity and negative predictive value were obtained for oxidized LDL in predicting pathological PWV.

Pulse pressure amplification, the ratio between central and peripheral pulse pressure, is related to an increase of pulse pressure (PP) amplitude as pressure waves propagate distally in the vessel network and is a measure of arterial elasticity [50, 51]. Changes in PP amplification are associated with traditional cardiovascular risk factors, including hypertension [52]. In our study, oxLDL, elevated hsCRP, and LDL values significantly correlated with PP amplification.

The most important study limitations include the cross-sectional study design, which does not provide any data about the cause-effect relationship, the relatively small sample size, and not considering the duration of daily sun exposure, which is important for vitamin D synthesis. Further larger studies are needed to demonstrate if vitamin D level, hsCRP, LDL, and oxidized LDL play important roles in the development of increased arterial stiffness and early arterial aging in hypertensive patients and to reveal their relationship with cardiovascular events, such as stroke and heart failure in hypertensive patients. A power analysis, using PASS 2019, was conducted before the study to determine the sample size needed for the present study, and we do not expect changes in the results by increasing the sample size. A minimum sample size required for Bravais-Pearson's and Spearman's correlations was 35 participants (desired statistical power: 0.8; correlation coefficient: 0.4). The required sample size for an area under the curve (AUC) of 0.7 and power and alpha of 0.75 and 0.15, respectively, was 52. One reading was made for each study participant for PWV, AI, and AP using the Mobil-O-Graph, which might be considered as another study limitation; however, earlier measurements provided by the same device demonstrated good reproducibility. According to Table 1, average blood pressure values are not impressive (137/91 mmHg) and PWV is not very high (on average 7.26 m/s). However, the population is proper for the study purpose, considering that PWV was increased for age in 21.4% of the participants and high normal and grade 1-3 hypertension patients were included, with both high and low cardiovascular risks. The cut-off values for increased PWV were provided by the Mobil-O-Graph considering age, and they were lower than those recommended by the European Guidelines (a threshold of 10 m/s for pathological values) [53]. The latter should emphasize the prophylactic character of the present study.

Our study is the first one comparing the predictive value of vitamin D, LDL, oxidized LDL, and hsCRP for arterial stiffness and early arterial aging, demonstrating a high sensitivity only for hsCRP, and revealing, for the first time, as far as we know, a correlation between pulse pressure amplification and oxidized LDL level. The findings of the present study have clinical implications, considering the prognostic importance of increased arterial stiffness and early arterial aging for cardiovascular risk. hsCRP, LDL, and oxidized LDL can provide valuable information related especially to large vessels, arterial age, and cardiovascular risk in treated hypertensive patients.

Hypertension is a state of low vitamin D level, oxidative stress, and low-grade inflammation. Further larger follow-up studies are needed in hypertensive patients to confirm the findings of the present study and to correlate the collagen content of the arterial wall with vitamin D level, high-sensitivity C-reactive protein, LDL, and oxidized LDL level, in order to confirm their role as cardiovascular active substances.

## 5. Conclusions

Concluding, low vitamin D levels and increased hsCRP and LDL values are very common in middle-aged hypertensive and high normal blood pressure patients, and they provide, along with oxidized LDL, valuable information about vascular function. However, only high-sensitivity C-reactive protein level is a sensitive predictor of increased arterial stiffness and early arterial aging.

## Figures and Tables

**Table 1:** Demographical and clinical characteristics of the study population (*n* = 56).

Characteristics	Means ± SD
Age (years)	48 ± 6

Gender (male)	32 (57%)

BMI (kg/m^2^)	27.14 ± 6.01

SBP (mmHg)	137 ± 14

DBP (mmHg)	91 ± 11

MAP (mmHg)	111 ± 11

Heart rate (beats/minute)	69 ± 11

PP (mmHg)	45 ± 10

cSYS (mmHg)	127 ± 13

cDIA (mmHg)	93 ± 11

cPP (mmHg)	35 ± 9

TVR (s × mmHg/ml)	1.28 ± 0.18

Augmentation pressure (mmHg)	7.9 ± 6.5

Augmentation index (AI) (%)	20 ± 13.71

Pulse wave velocity (PWV) (m/s)	7.26 ± 0.69

PWV increased for age (pathological PWV)	12 (21.4%)

Early arterial aging	18 (32.1%)

Family history of cardiovascular disorders	13 (22.5%)

Cardiovascular risk	High: 17 (30%)
	Low: 14 (25%)

Hypertension (HT)	High normal: 21 (37.5%)
	HT grade 1: 16 (28.6%)
	HT grade 2: 13 (23.2%)
	HT grade 3: 6 (10.7%)

Therapy	ACEI: 17 (30%)
	Sartans: 10 (17.5%)
	Diuretics: 18 (32.5%)
	Beta blockers: 18 (32.5%)
	Calcium channel blockers: 4 (7.5%)

BMI=body mass index; SBP=systolic blood pressure; DBP=diastolic blood pressure; MAP=mean arterial pressure; PP=pulse pressure; cSYS=central systolic blood pressure; cDIA=central diastolic blood pressure; cPP=central pulse pressure; TVR=total vascular resistance; ACEI=angiotensin converting enzyme inhibitors; HT=hypertension.

**Table 2:** Biomarkers.

Biomarker	Means ± SD
Vitamin D (microg/l)	25.99 ± 11.17
Vitamin D < 20 microg/l	19 (34%)
Vitamin D < 30 microg/l	41 (73%)
hsCRP (mg/dl)	0.48 ± 0.44
hsCRP > 0.1 mg/dl	43 (77%)
hsCRP > 0.3 mg/dl	29 (52%)
LDL (mg/dl)	145.73 ± 39.53
LDL > 100 mg/dl	49 (88%)
LDL > 130 mg/dl	35 (62.5%)
LDL > 160 mg/dl	17 (30%)
Oxidized LDL (ng/ml)	261.37 ± 421

LDL=low-density lipoprotein; hsCRP=high-sensitivity C-reactive protein.

**Table 3:** Correlations between variables of central hemodynamics and pulse wave analysis with high-sensitivity C-reactive protein (hsCRP), LDL, and oxidized LDL (oxLDL) cholesterol, respectively.

Correlated variables	Correlation coefficient	*p*
hsCRP-EAA	rP = 0.376	0.004
	rK = 0.332	0.003
	rS = 0.402	0.002
hsCRP-PWV	rK = 0.225	0.016
	rS = 0.308	0.021
hsCRP-pPWV	rP = 0.398	0.002
	rS = 0.378	0.004
hsCRP-EAA	rP = 0.358	0.007
CRP100-PWV	rP = 0.283	0.031
	rK = 0.242	0.032
	rS = 0.288	0.031
CRP100-cPP/PP	rP = 0.288	0.031
CRP100-EAA	rS = 0.288	0.031
CRP300-PWV	rP = 0.351	0.008
	rK = 0.357	0.002
	rS = 0.425	0.001
CRP300-EAA	rP = 0.358	0.007
	rK = 0.358	0.008
	rS = 0.358	0.007
LDL100-PWV	rP = 0.306	0.022
LDL130-cSYS	rP = −0.311	0.020
	rK = −0.248	0.028
	rS = −0.297	0.026
LDL130-cDIA	rP = −0.276	0.039
LDL130-PWV	rP = 0.303	0.023
LDL130-cPP/PP	rP = −0.285	0.033
oxLDL-cPP/PP	rK = −0.230	0.042
	rS = −0.347	0.028

rP=Bravais-Pearson's correlation coefficient; rK=Kendall's correlation; rS=Spearman's correlation; cPP/PP=pulse pressure amplification; EAA=early arterial aging; PWV=pulse wave velocity; pPWV=pathological pulse wave velocity (increased for age); cSYS=central systolic blood pressure; cDIA=central diastolic blood pressure; hsCRP=high-sensitivity C-reactive protein; CRP100=high-sensitivity C-reactive protein exceeding 0.100 mg/dl; CRP300=high − sensitivity C − reactive protein > 0.300 mg/dl; LDL100=LDL > 100 mg/dl; LDL130=LDL > 130 mg/dl; LDL160=LDL > 160 mg/dl; oxLDL=oxidized LDL.

**Table 4:** Results of receiver-operating characteristic (ROC) curve analysis for high-sensitivity C-reactive protein (hsCRP).

Test variable	State variable	AUC (95% CI)	*p*	Cut-off value	Sensitivity	Specificity
hsCRP	EAA	0.749 (0.607-0.890)	0.003	0.388	83.3%	71.1%
hsCRP	pPWV	0.766 (0.603-0.929)	0.005	0.446	83.3%	65.6%

EAA=early arterial aging; PWV=pulse wave velocity; pPWV=pathological pulse wave velocity (increased for age).

**Table 5:** Results of receiver-operating characteristic (ROC) curve analysis for vitamin D, LDL, and oxidized LDL as predictors of pathological pulse wave velocity and early arterial aging.

Test variable	State variable	AUC (95% CI)	*p*
Vitamin D	pPWV	0.473 (0.274-0.673)	0.78
Vitamin D	EAA	0.487 (0.324-0.65)	0.875
LDL	pPWV	0.393 (0.227-0.559)	0.259
LDL	EAA	0.45 (0.291-0.608)	0.545
oxLDL	pPWV	0.547 (0.314-0.78)	0.685
oxLDL	EAA	0.484 (0.292-0.676)	0.874

pPWV=pathological pulse wave velocity (increased for age); EAA=early arterial aging; LDL=low-density lipoprotein; oxLDL=oxidized LDL.

**Table 6:** Sensitivity, specificity, and positive and negative predictive values.

Test variables	Sensitivity (95% CI)	Specificity (95% CI)	Positive predictive value (PPV) (95% CI)	Negative predictive value (NPV) (95% CI)
D20-pPWV	0.363 (0.123-0.683)	0.666 (0.509-0.795)	0.21 (0.069-0.46)	**0.81** (0.642-0.914)
D20-EAA	0.333 (0.143-0.588)	0.657 (0.485-0.798)	0.315 (0.135-0.565)	0.675 (0.501-0.814)
LDL160-pPWV	0.055 (0.002-0.293)	0.605 (0.434-0.755)	0.062 (0.003-0.322)	0.575 (0.41-0.725)
LDL160-EAA	0.166 (0.04-0.422)	0.631 (0.459-0.777)	0.176 (0.046-0.441)	0.615 (0.446-0.761)
oxLDL-pPWV	0.25 (0.044-0.644)	**0.843** (0.664-0.941)	0.285 (0.051-0.697)	**0.818** (0.639-0.923)
oxLDL-EAA	0.153 (0.027-0.463)	**0.814** (0.612-0.929)	0.285 (0.051-0.697)	0.666 (0.481-0.814)
CRP100-pPWV	**0.833** (0.508-0.97)	0.272 (0.154-0.43)	0.238 (0.125-0.398)	**0.857** (0.561-0.974)
CRP100-EAA	**0.944** (0.706-0.997)	0.315 (0.18-0.487)	0.395 (0.253-0.555)	**0.923** (0.62-0.995)
CRP300-pPWV	0.75 (0.428-0.933)	0.545 (0.389-0.693)	0.31 (0.159-0.509)	**0.888** (0.697-0.97)
CRP300-EAA	0777 (0.519-0.926)	0.605 (0.434-0.755)	0.482 (0.298-0.671)	**0.851** (0.653-0.951)

pPWV=pathological pulse wave velocity (increased for age); EAA=early arterial aging; oxLDL=oxidized LDL; LDL160=LDL > 160 mg/dl; CRP100=high-sensitivity C-reactive protein exceeding 0.100 mg/dl; CRP300=high-sensitivity C-reactive protein > 0.300 mg/dl; D20=vitamin D < 20 microg/l. The highest values for sensitivity, specificity, and NPV are in bold.

## Data Availability

The data used to support the findings of this study are available from the corresponding author upon request.
